# Fragment synthesis of disulfide-containing peptides

**DOI:** 10.1016/j.mex.2020.100945

**Published:** 2020-06-04

**Authors:** Yuxuan Dai, Na Yue, Wenlong Huang, Hai Qian

**Affiliations:** a Center of Drug Discovery, State Key Laboratory of Natural Medicines, China Pharmaceutical University, 24 Tongjiaxiang, Nanjing 210009, PR China; b Jiangsu Key Laboratory of Drug Discovery for Metabolic Disease, China Pharmaceutical University, 24 Tongjiaxiang, Nanjing 210009, PR China

**Keywords:** Peptide conjugate, Fragment synthesis, Solid phase synthesis

## Abstract

A new strategy for solid phase peptide synthesis in the fragment synthesis based on the use of 2-Cl-trityl resin as carrier to obtain protected peptide-resin and Rink Amide Resin, HOBT/HBTU or PyBOP/DIPEA or HATU/DIPEA as the coupling method is described. The highlights of this method are:

• Simple.
• Low cost.
• Solid phase based.

## Graphical abstract

**Figure:**
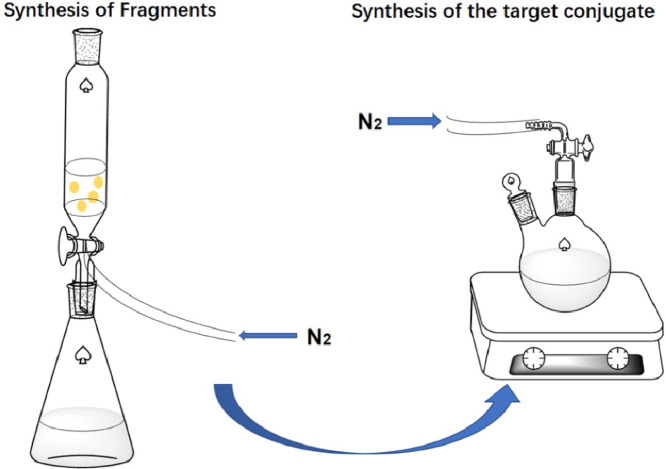

Specifications Table

**Table:** 

Subject Area Chemistry	Select one of the following subject areas: • Agricultural and Biological Sciences • Biochemistry, Genetics and Molecular Biology • Chemical Engineering • Chemistry • Computer Science • Earth and Planetary Sciences • Energy • Engineering • Environmental Science • Immunology and Microbiology • Materials Science • Mathematics • Medicine and Dentistry • Neuroscience • Pharmacology, Toxicology and Pharmaceutical Science • Physics and Astronomy • Psychology • Social Sciences • Veterinary Science and Veterinary Medicine
More specific subject area: Organic chemistry	Describe narrower subject area
Method name: Fragment synthesis of peptides	Please specify a name of the method that you customized. The method name should be a word or short phrase to describe the methods used in your paper
Name and reference of original method	If applicable, include full bibliographic details of the main reference(s) describing the original method from which the new method was derived.
Resource availability	If applicable, include links to resources necessary to reproduce the method (e.g. data, software, hardware, reagent)

*Method details.

## Materials

All reagents were purchased as reagent grade and used without further purification. N^α^-Protected amino acids, Fmoc-Arg(Pdf)-OH, Fmoc-Leu-OH, Fmoc-Gly-OH, Fmoc-Pro-OH, Fmoc-Trp (Boc)-OH, Fmoc-_d_-Trp (Boc)-OH, 2-Cl-trityl resin (loading 0.85 mmol g^_1^) and Rink amide MBHA resin (loading 0.557 mmol g^_1^), were purchased from GL Biochem Ltd. (Shanghai, China). Fmoc-Ser(tBu)-OH, Fmoc-Lys (Boc)-OH, Fmoc-Phe-OH, Fmoc-His(Trt)-OH and N—Hydroxybenzotriazole (HOBt), were purchased from Nanjing Peptide Biotech Ltd. N, N-diisopropylcarbodiimide (DIC), trifluoroacetic acid (TFA), trifluoroethanol (TFE) and N, N-diisopropylethyl amine (DIPEA) were purchased from Energy chemical. Paclitaxel was purchased from Ark Pharm. Pteroic acid and 2-(7-Azabenzotriazol-1-yl)-N,N,N',N'-tetramethyluronium hexafluorophosphatewas (HATU) purchased from Bide Pharmatech. Cystamine dihydrochloride was purchased from Aladdin. All other reagents, unless otherwise indicated, were obtained from Sigma-Aldrich Co. (Saint Louis, MO).

## Method overview

Peptide synthesis is a process of repeating the addition of amino acids^[^^1^^]^. Synthesis is generally synthesized from the C-terminus (carboxy terminus) to the N-terminus (amino terminus). Since Merrifield developed a solid phase peptide synthesis method in 1963, it has been continuously improved and perfected ^[^^1^^,^^2^^]^. Solid phase method has become a common technique in peptide and protein synthesis and shows the advantages over classical liquid phase synthesis ^[^[3], [4], [5]^]^. From the first amino acid to the last solid-phase synthesis was used widely to prepare peptides^[^[6], [7], [8]^]^. However, the method was limited because its purity has been declining as the synthesis progresses ^[^^9^^,^^10^^]^ .

**Fig. 1:**
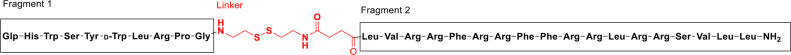
The structure of target conjugate.

A new strategy for solid phase peptide synthesis in the fragment synthesis based on the use of 2-Cl-trityl resin as carrier to obtain protected peptide-resin and Rink Amide Resin, HOBT/HBTU or PyBOP/DIPEA or HATU/DIPEA as the coupling method is described. In this study, we introduced a fragment synthesis method of target conjugate (Fig. 1). The fragment synthesis method was adopted for increasing the reaction efficiency and decreasing the use of reagents.

Fragment 1 and Fragment 2 were synthesized using standard 9-fluorenylmethoxycarbonyl (Fmoc) solid phase synthesis techniques.

## Method description

### Synthesis of Fragment 1

**Fig. 2:**
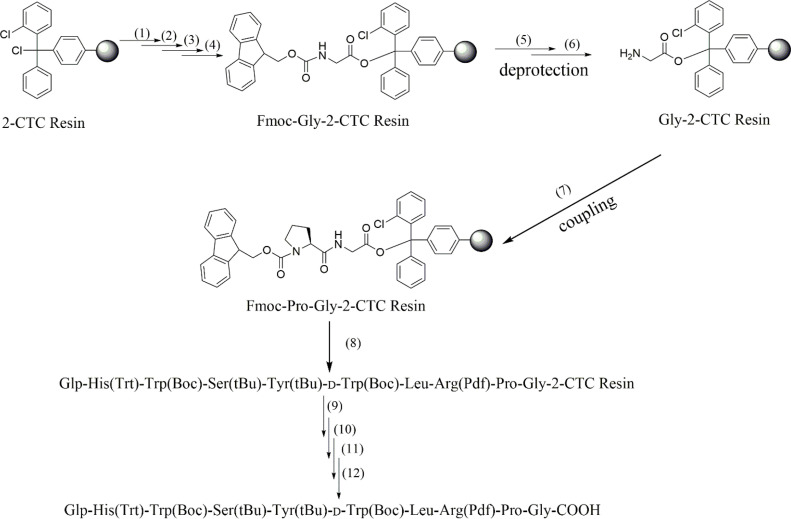
Synthetic route of Fragment 1 with protecting groups.

Fragment 1 was prepared on 2-Cl-trityl resin. Synthetic route of Fragment 1 was shown in Fig. 2.

Progress one:1, Place the 2-Cl-trityl resin (1 mmol, 1.18 g, degree of substitution 0.85 mmol/g) in a glass funnel and swell the resin with DCM (30 ml) for 30 min; 2, Weigh out Fmoc-Gly-OH (5 eq), DIPEA (10 eq); 3, Add 15 mL of DCM to effect total dissolution and blow nitrogen; 4, Agitate resin gently for 1 h; 5, Deprotection 0.1 mmol/L HOBt, 20% Piperidine/DMF (V/V), two times; 6, Wash resin with DMF(4 × 20 ml); 7, Weigh out Fmoc-amino acid (5 eq), HOBT (5 eq) and HBTU (5 eq) and 18 mL of DMF to effect total dissolution, add DIPEA (10 eq) and blow nitrogen for 2 h; 8,Repeat steps 5–7; 9, Cleavage of protected peptide fragment from 2-Cl-trityl resin. Place 1 g peptide-resin in a flask, add 15 ml of TFE/DCM (2:8) cleavage solution for 45 min; 10. Concentrate the filtrates and add 20 ml cold ether and 10 ml hexane; 11.Centrifugate and collect the resulting solid and wash with ether (4 × 10 ml); 12. Dry the protected peptide in vacuum and store at −20 °C (Fragment 1).

### Synthesis of linker

**Fig. 3:**
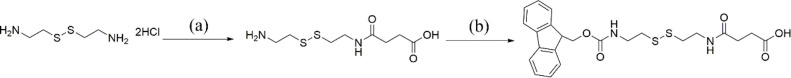
Synthetic route of Linker (Fmoc-cystamine succinic acid) for solid phase peptide. (a) Succinic anhydride, NaHCO3, 1,4-Dioxane/H2O, 0 °C; (b) Fmoc-OSu, DMF, 0 °C.

Fig. 3 shown the synthesis of Linker.

Progress two:Cystamine dihydrochloride (0.45 g, 2 mmol) and NaHCO_3_ (0.5 g, 6 mmol) was dissolved in 2 ml H_2_O and 20 ml Dioxane. Succinic anhydride (0.2 g, 0.4 mmol) was added to the above mixture in the ice bath. The resulting reaction mixture was stirred overnight. Then, 0.33 mL of DIPEA and (0.67 g, 2 mmol) of Fmoc-OSu were added to the reaction mixture at 0 °C. The reaction mixture was stirred for 4 h. Then, the filtrate was concentrated under reduced pressure. 40 mL of water was added. the white precipitate was collected by filtration, and dried in vacuum, sequentially. 0.34 g of Fmoc-cystamine succinic acid (Fmoc-CS) was acquired by column chromatography (DCM: Methanol, 20:1) with a yield of 36%. Fmoc-CS, white solid; yield, 36%. 1H NMR (300 MHz, DMSO‑d6) δ 8.14 (s, 1H, NH); δ 7.88–7.90 (d, *J* = 6.00 Hz, 2H, ArH); 7.68–7.70 (d, *J* = 6.00 Hz, 2H, ArH); 7.39–7.44 (t, 2H, ArH); 7.31–7.35 (m, 2H, ArH); 6.28 (s, 1H, NH);4.30–4.32 (d, *J* = 6.30 Hz, 2H, OCH_2_); 4.22–4.23 (m, 1H, CH); 3.06–3.34 (m, 4H, 2 × CH_2_); 2.50–2.76 (m, 4H, 2 × SCH_2_); 2.23–2.35 (m, 4H, 2 × CH_2_). MS (ESI) *m/z* calcd for [C_32_H_26_N_2_O_5_S_2_ + Na]^+^ 497.6, found 497.7.

### Synthesis of Fragment 2

**Fig. 4:**
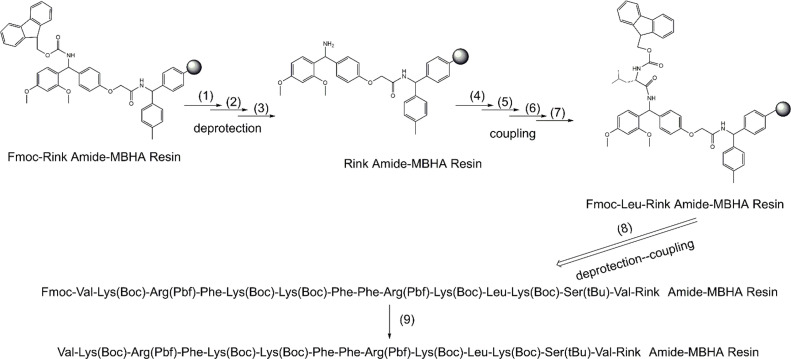
Synthetic route of Fragment 2 with protecting groups.

Fragment 2 was prepared on Rink Amide MBHA resin. Synthetic route of Fragment 2 was shown in Fig. 4.

Progress three:1, Place the Rink Amide MBHA resin in a glass funnel and swell the resin with DCM (30 ml) for 30 min; 2, Deprotection: 0.1 mmol/L HOBt, 20% Piperidine/DMF (V/V), two times; 3, Wash resin with DMF(4 × 20 ml); 4. Weigh out Fmoc-amino acid (5 eq), HOBT (5 eq) and HBTU (5 eq); 5, Add 18 mL DMF to effect total dissolution; 6, Add DIPEA (10 eq) and blow nitrogen; 7, Agitate resin gently for 2 h; 8, Repeat steps 2–6; 9, Collect the peptide-resin (Fragment 2).

### Synthesis of the target conjugate

**Fig. 5:**
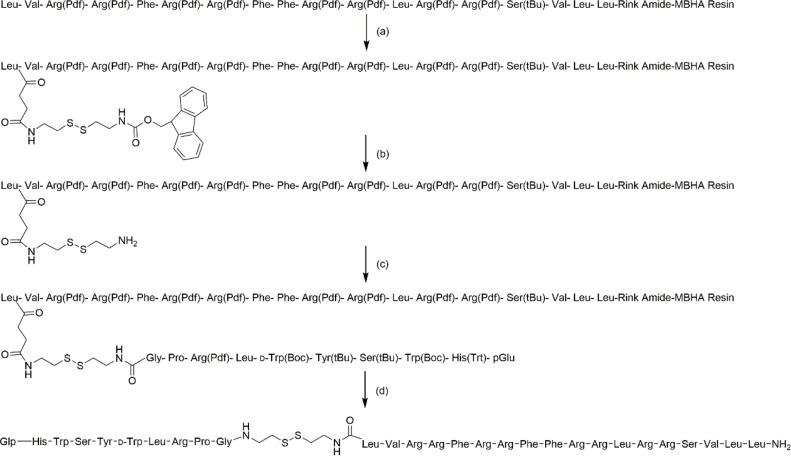
Synthetic route of target conjugate. a) Fmoc-CS, PyBop, DIPEA, DMF; (b) 0.1 mmol/L HOBt, 20% Piperidine/DMF (V/V); (c) Glp-His(Trt)-Trp(Boc)-Ser(tBu)-Tyr(tBu)-DTrp(Boc)-Leu-Arg(Pdf)-Pro-Gly-COOH, HATU, DIPEA, DMF; (d) TIS, H2O, TFA.

Synthetic route and MS data of target conjugate was shown in Fig. 5 and Table 1, respectively.

**Table 1:** MS data of the target conjugate.

		Mass (Da)			
Compounds	Molecular weight	Calculated		Observed	
target conjugate	3974.8	[*M* + 4H]^4+^	994.7	[*M* + 4H]^4+^	995.3
		[*M* + 5H]^5+^	796.0	[*M* + 5H]^5+^	796.5

Progress four:1, Place the Fragment 2 into a glass funnel and swell the resin with DCM(30 mL) for 30 min; 2, Add the Linker (2 eq), PyBOP (3 eq) and DIPEA (3 eq); 3, Add 16 mL of DMF to effect total dissolution and blow nitrogen for 4 h; 4, Wash resin with DMF(3 × 20 ml); 5. Deprotection: 0.1 mmol/L HOBt, 20% Piperidine/DMF (V/V), two times; 6. wash resin with DMF(4 × 20 ml); 7, Add Fragment 1(2 eq), HATU (2eq) and DIPEA(4 eq); 8, Add 14 mL of DMF to effect total dissolution and blow nitrogen for 4 h; 9. wash resin with DMF(3 × 20 ml) and DCM(2 × 20 ml); 10, Cleavage of target conjugate: TIS: H_2_O: TFA (2.5:2.5:95). 11, Concentrate the filtrates and add 40 ml cold ether, collect the solid and wash the solid with cold ether four times; 12, Dry the target peptide in vacuum and store at −20 °C.

## Declaration of Competing Interest

The authors have no conflicts of interest to declare.
